# Lung Ultrasound Reduces the Number of Chest x-rays in Newborns with Pneumothorax

**DOI:** 10.34763/devperiodmed.20192303.172177

**Published:** 2019-10-27

**Authors:** Izabela Szymońska, Łukasz Wentrys, Mateusz Jagła, Marta Olszewska, Weronika Wasilewska, Barbara Smykla, Przemko Kwinta

**Affiliations:** 1 Department of Pediatrics, Jagiellonian University Collegium Medicum, Kraków, Poland

**Keywords:** pneumothorax, newborns, lung ultrasound, odma, noworodki, ultrasonografia płuc

## Abstract

**Aim of the study:**

To determine the impact of lung ultrasonography as an imaging method used to diagnose and monitor newborns with symptomatic pneumothorax and to assess the risk factors for pneumothorax and the outcomes in newborns with symptomatic pneumothorax.

**Material and methods:**

A single-centre retrospective study enrolled patients born after 32 weeks of gestation, with a diagnosis of pneumothorax in the first week of life. The 118 patients who were included in the study were divided into two groups. Group A (51 infants) comprised those children who were treated between 2007 and 2010, while group B (n=67) those from the years 2013 to 2016. The children from group A were monitored with repeated chest X-rays. Those from group B received repeated lung ultrasonography supported by chest X-ray in those cases where there was diagnostic uncertainty. Comparison was made between the groups with respect to pneumothorax risk factors, treatment methods and the use of imaging during the period of treatment. The statistical analysis used χ2, Mann-Whitney and Student’s t-tests.

**Results:**

There were no significant demographic or clinical differences between the two groups. Both the use of nCPAP (nasal continuous positive airway pressure) (p<0.001) and diagnosed perinatal asphyxia (p=0.036) were higher in group B. Congenital pneumonia occurred more often in group A (p=0.041). Earlier detection of pneumothorax (p=0.001) and shorter hospital stay (p=0.03) were observed in group B. However, the total number of imaging (lung ultrasound and chest X-ray combined) was higher (p<0.001) in group B.

**Conclusion:**

This study confirmed the usefulness of lung ultrasound in monitoring newborns with pneumothorax, moreover significantly limiting X-ray radiation.

## Streszczenie

**Cel:**

Określenie znaczenia ultrasonografii płuc jako metody obrazowania wykorzystywanej w diagnostyce i monitorowaniu objawowej odmy opłucnowej u noworodków. Ocena czynników związanych z występowaniem odmy opłucnowej oraz rokowania w grupach noworodków z odmą opłucnową objawową.

**Materiał i metody:**

Do retrospektywnego, jednoośrodkowego badania włączono noworodki urodzone po 32 tygodniu ciąży, u których zdiagnozowano odmę opłucnową w pierwszym tygodniu życia. Do badania zakwalifikowano 118 pacjentów. Grupa A obejmowała 51 pacjentów z okresu od 2007 do 2010 roku. Grupa B liczyła 67 pacjentów z okresu od 2013 do 2016 roku. Noworodki z grupy A były monitorowane za pomocą powtarzanych zdjęć rentgenowskich klatki piersiowej. Noworodki w grupie B kontrolowano powtarzanymi badaniami ultrasonograficznymi płuc, zdjęcia rentgenowskie klatki piersiowej wykonywano w przypadku niepewności diagnostycznej. Porównano obydwie grupy pod względem czynników ryzyka, metod leczenia oraz technik obrazowania w okresie terapii. Do analizy statystycznej użyto testów χ2, Mann-Whitney i t-Studenta.

**Wyniki:**

Grupy nie różniły się w sposób istotny pod względem danych demograficznych i klinicznych. W grupie B częściej stosowano nCPAP (p<0,001) oraz częściej diagnozowano niedotlenienie okołoporodowe (p=0,036). Wrodzone zapalenie płuc częściej rozpoznawano w grupie A (p=0,041). Wcześniejsze wykrycie odmy (p=0,001) oraz z krótszy czas hospitalizacji (p=0,03) obserwowano w grupie B. Istotnie mniej zdjęć rentgenowskich klatki piersiowej zostało wykonanych w grupie B. Jednakże, sumaryczna liczba wykonanych badań (ultrasonografii płuc i zdjęć rentgenowskich klatki piersiowej) była wyższa (p<0,001) w grupie B.

**Wnioski:**

Badanie potwierdziło korzyści z zastosowania ultrasonografii płuc w procesie diagnostyczno--leczniczym noworodków z odmą opłucnową oraz istotne zmniejszenie narażenia na promieniowanie rentgenowskie.

## Introduction

Pneumothorax (PTX) is defined as a collection of air between the parietal and visceral pleura. It is one of the most frequent emergencies in the newborn period, typically occurring in the first 2-3 days of life [1]. Its prevalence varies depending on gestational age (GA), ranging from 4.0% in early preterm, 2.6% in moderate-late preterm to 6.7% in term neonates [2]. Major risk factors for neonatal pneumothorax are: underlying pulmonary disease, ventilatory support, meconium aspiration syndrome (MAS), active resuscitation, male sex, large for gestational age (LGA) and high transpulmonary pressure generated at the onset of respiration [3, 4]. Many newborns with PTX have no symptoms, but some develop a life-threatening condition manifested by rapidly worsening symptoms of dyspnea. Tension PTX leads to increased intrathoracic pressure, elevated central venous pressure and decreased venous return. This can result in reduced cardiac output, bradycardia, and ultimately cardiac arrest [1, 5]. Traditionally pneumothorax is diagnosed by chest X-ray (CTX), which remains the gold-standard imaging test. However, chest X-rays expose children to radiation and diagnosis of small pneumothoraxes can be challenging [6, 7]. Lung ultrasonography (LUS) is a relatively new technique which has revolutionized lung imaging diagnostics and is gaining popularity [8]. Diagnostic markers for PTX can be detected using ultrasound with a high degree of accuracy; LUS has been shown to have both high sensitivity and specificity for diagnosing pneumothorax in newborns [6, 9]. The safety of LUS is supported by previous clinical studies [10]. In addition, ultrasound has the advantage being more rapidly available, performed at the bedside, lacking in radiation and potentially more accurate. Moreover, chest transillumination (CTR) allows rapid detection of PTX at the bedside. However, this method has its limits due to the possibility of false positive or negative results and is less accurate than CTX and LUS [11].

## Aim of the study

To determine the impact of lung ultrasonography as an imaging method used to diagnose and monitor newborns with symptomatic pneumothorax. To assess the risk factors for pneumothorax and the outcomes in newborns with symptomatic pneumothorax.

## Material and methods

A single-centre retrospective study was conducted at the Neonatal Intensive Care Unit of the Department of Pediatrics, Jagiellonian University, a tertiary level neonatal centre in Cracow, Poland.

Newborns included in the study were born after 32 weeks of gestation and were diagnosed with PTX in the first week of life. Those with major congenital malformations were excluded. 118 patients were enrolled in the study. All of them had clinical signs of respiratory distress. In all the cases, the diagnosis of PTX was confirmed by a radiologist after CXR. The patients were divided into two groups depending on their birthdate; Group A included 51 infants born from 2007 to 2010, while in group B there were 67 children born from 2013 to 2016. The patients delivered in 2011 and 2012 were not included in the study, as this time represents the training period for LUS and the development of care protocols for pneumothorax within the unit.

In Group A the children were monitored with repeated CXR, whereas in Group B with repeated LUS supported by CXR in those cases where there was diagnostic uncertainty. All the CXRs were validated by a radiologist. LUS examinations were performed using a linear high frequency probe (5-12 MHz, Envisor HD 11, Philips, USA).

LUS diagnosis was based on recognized sonographic signs of pneumothorax including: the presence of lung point(s), the absence of lung sliding, the absence of B-lines and the absence of lung pulse [5, 6, 7, 8, 12]. All of the ultrasonography was performed by neonatologists certified by the Polish Society of Ultrasonography.

All the patients required treatment of PTX, the options of which were: drainage by needle aspiration, needle aspiration followed by chest drain, or chest drain alone. The monitoring protocol consisted of observation during the period of active drainage. CXR (group A) or LUS (group B) were performed 12 hours after the cessation of drainage to evaluate PTX. In the case of re-accumulation of air, the drainage was opened and PTX reassessed.

The two groups were compared in terms of risk factors for PTX, such as maternal carriage of Group B streptococci (GBS), delivery method, need for delivery room cardiopulmonary resuscitation (CPR), mechanical ventilation (MV), respiratory distress syndrome (RDS), meconium aspiration syndrome (MAS), lung hypoplasia, congenital pneumonia and congenital lung disorders. Moreover, the duration of drainage, length of hospitalization and morbidity were compared between the groups.

As regards statistical analysis, χ2, Mann-Whitney and Student’s t-tests were used to compare baseline and outcome variables, as appropriate. The results are presented as a number (percentage) or median (interquartile range), unless otherwise indicated. Probability values below 0.05 were considered statistically significant. MedCalc Statistical Soſtware version 14.12.0 was used for statistical analysis (MedCalc Soſtware bvba, Ostend, Belgium; http://www.medcalc.org; 2014).

## Results

The demographic and clinical characteristics of the patients are presented in Table 1. No significant differences were found between the groups. The comparison of risk factors for pneumothorax is also presented in Table I.

**Table I:** Demographic and clinical characteristics of the patients and risk factors. Tabela I. Dane demograficzne, charakterystyka kliniczna pacjentów oraz czynniki ryzyka.

	Group A *Grupa A* n=51	Group B *Grupa B* n=67	P-value *Wartość p*
GA, weeks *Wiek płodowy, tygodnie* (IQR)	37 (32.25-38.75)	37 (35-38)	0.53^b^
Birth weight, g *Masa urodzeniowa*, g (SD)	2876.0±731.0	2944.0±659.0	0.60^c^
Male, n *Płeć męska* n (%)	18 (35,3)	21 (30,9)	0.84^a^
Apgar Score 1 min score n 1 min score, n *Punktacja w Skali Apgar w 1 minucie*, n (%)	8 (6-9)	9 (7-10)	0.52^b^
Apgar score 5 min score, n *Punktacja w Skali Apgar w 5 minucie*, n (%)	8 (7-9)	9 (8-9)	0.07^b^
Antenatal steroids, n *Podaż sterydów prenatalnie*, n (%)	1 (2,0)	8 (11,9)	0.08^a^
Neopuff, n *Neopuff, n (%)*	9 (17.6)	6 (9.0)	0.17^a^
Endotracheal tube, n *Intubacja dotchawicza*, n (%)	7 (13.7)	10 (14.9)	1.0^a^
nCPAP therapy, n *Terapia nCPAP*, n (%)	14 (27.5)	50 (74.6)	<0.001^a^
SIMV, n *Wentylacja mechaniczna, n (%)*	22 (43.2)	40 (56.8)	0.09^a^
RDS, n *Zespół zaburzeń oddychania, n* (%)	6 (11.8)	9 (13.4)	1.0^a^
Surfactant therapy, n *Terapia surfaktantem*, n (%)	7 (13.7)	13 (19.7)	0.46^a^
Congenital pneumonia, *Wrodzone zapalenie płuc*, n (%)	32 (62.7)	29 (43.3)	0.041^a^
MAS, n *Zespół aspiracji smółki, n* (%)	4 (7.8)	3 (4.5)	0.46^a^
Lung hypoplasia, *Hipoplazja płuc*, n (%)	0 (0.0)	2 (3.0)	0.51^a^
Congenial lung abnormalities, *Wrodzone wady płuc*, n (%)	2 (3.9)	2 (3.0)	1.0^a^
Perinatal asphyxia, *Niedotlenienie okołoporodowe*, n (%)	0 (0.0)	6 (9.0)	0.036^a^

Data are number (%), mean (±SD) or median (IQR). P value: a is for χ2 test, b is for Mann-Whitney, c is for Student’s t-test. GA: gestational age. nCPAP: Nasal Continuous Positive Airway Pressure, SIMV: Synchronized Intermittent Mandatory Ventilation, RDS: Respiratory Distress Syndrome, MAS: Meconium Aspiration Syndrome. In the delivery room: Neopuff, endotracheal tube During hospitalization: nCPAP therapy, SIMV. *Dane są przedstawione jako procent (%), średnia (±SD) lub mediana (IQR). Wartość p: a dla testu χ2, b dla testu Mann-Whitney, c dla* *testu Student. GA: wiek ciążowy. nCPAP: ciągłe dodatnie ciśnienie w drogach oddechowych metodą donosową, SIMV: synchronizowana przerywana wentylacja obowiązkowa, RDS: zespół zaburzeń oddychania, MAS: zespół aspiracji smółki. Na sali porodowej: Neopuff, intubacja dotchawicza W trakcie hospitalizacji: terapia nCPAP, SIMV*.

When considering the methods of respiratory support, no differences were found between the use of Neopuff or intubation in the delivery room or in Positive Pressure Ventilation during hospitalization. However, nasal continuous positive airway pressure (nCPAP) was more common in group B (p<0.001). Comorbidities, such as RDS, MAS, lung hypoplasia and congenital lung abnormalities were not significantly different between the groups. However, perinatal asphyxia (p=0.036) was diagnosed more often in group B and congenital pneumonia occurred more frequently in group A (p=0.041).

The pneumothorax characteristics, its clinical course, and imaging modalities used in each group are presented in Table II. PTX was detected earlier in Group B (p=0.001). In group A there were 4 infants who were diagnosed with pneumopericardium in contrast to none in group B. The most important difference was seen in the monitoring of the pneumothorax, with significantly fewer CTX performed following the introduction of the new imaging method. Interestingly, the total number of all the images performed (CXR and LUS together) was higher in Group B (p<0.001). Finally, hospital stay was significantly shorter in Group B (p=0.03).

**Table II:** Pneumothorax characteristics, imaging studies and clinical course in the two groups. Tabela II. Charakterystyka odmy opłucnowej, zastosowanych badań obrazowych i przebiegu klinicznego w grupach.

	Group A *Grupa A* n=51	Group B *Grupa B* n=67	P-value *Wartość p*
Pneumothorax characteristics *Charakterystyka odmy opłucnowej*			
Diagnosis DOL, *Diagnoza, doba życia*, n (IQR)	2 (1-3)	1,5 (1-2)	0.001^b^
Bilateral, n *Obustronna*, n (%)	6 (11.8)	17 (25.4)	0.10^a^
Pneumomediastinum, *Odma śródpiersia*, n (%) n	9 (17.6)	6 (13.4)	0.61^a^
Pneumopericardium, n *Odma osierdziowa*, n (%)	4 (7.8)	0 (0.00)	0.03^a^
Imaging studies *Badania obrazowe*			
LUS per patient, n *Liczba usg na pacjenta, n (IQR)*	0	4 (3-5)	<0.0001^b^
CTX per patient, n *Liczba rtg na pacjenta, n (IQR)*	4 (3-5)	1 (1-2)	<0.0001^b^
LUS and CTX, n *Liczba usg i rtg łącznie, n (IQR)*	4 (3-5)	5 (5-6)	<0.001^b^
Clinical course *Przebieg kliniczny*			
Drainage days, n *Ilość dni drenażu*, n (IQR)	2 (0-4)	3 (1.75-4.0)	0.11^b^
MV days, n *Ilość dni mechanicznej wentylacji*, n (IQR)	1 (0-5)	2 (2-3)	0.33^b^
Hospitalization days, n *Dni hospitalizacji*, n (IQR)	15 (13-18)	11 (10-14)	0.03^b^
Death before discharge, n *Zgon przed wypisem*, n (%)	4 (7.8)	2 (3.0)	0.41^a^

Data are number (%) or median (IQR). P value: a is for χ2 test, b is for Mann-Whitney. DOL: day of life, LUS: lung ultrasonography, CTX: chest X-ray, MV: mechanical ventilation. *Dane są przedstawione jako procent (%) lub mediana (IQR). Wartość P: a dla testu χ2, b dla testu Mann-Whitney. DOL: doba życia, LUS*: *ultrasonografia płuc, CTX: zdjęcie rentgenowskie klatki piersiowej, MV: wentylacja mechaniczna*.

## Discussion

The appearance of brand-new diagnostic imaging techniques, such as lung ultrasound (LUS), opens new possibilities in neonatology. The use of ultrasound in neonates has revolutionized patient monitoring and treatment options, not only in the case of lung abnormalities. This study presents the impact of LUS on the clinical management of newborns with pneumothorax.

Despite the passage of time, PTX remains a serious newborn emergency. Its unpredictable appearance can lead to clinical destabilization and even death. Although common, the incidence of PTX among newborns varies among authors from 1.0% to as high as 7.6% [3, 13], depending on study indications, the treatment given and the gestational age of the patients.

For many years, CXR has been the gold standard imaging test for PTX diagnosis and monitoring. However, recently, LUS has gained in popularity. Following a short training course, LUS may be reliably performed by neonatal doctors at the bedside [9, 14]. LUS diagnostic accuracy for PTX has been confirmed in paediatric and adult population studies and can even reach 100% in sensitivity, specificity, positive and negative predictive value [6, 7]. The examination can be rapidly performed at the bedside, avoiding delay in diagnosis or treatment (point of care diagnosis) [12] and is radiation-free. LUS is particularly reliable in infants, because of their thinner chest wall, smaller thoracic width and lower lung mass [15]. However, there are limitations of this method, causing false positive or negative results, i.e. children with chest wall oedema, subcutaneous chest wall air, pneumomediastinum, or severe pulmonary interstitial emphysema; thick chest wall, darkly pigmented skin, or non-adequate light conditions, respectively [6, 15]. Moreover, CXR is also imperfect and can under-estimate a substantial proportion of cases, a phenomenon known as radio-occult PTX [12]. The growing acceptance of LUS has resulted in its being recommended over CTX in certain situations, including cardiac arrest, the unstable patient, radio-occult pneumothorax, and limited-resource areas [5, 8].

By comparing two distinct time intervals, our study presents the effect of this new diagnostic method on symptomatic PTX monitoring in newborns. Although demographically similar, there were statistical differences in PTX risk factors between the groups. Due to developments in neonatology, the incidence of nCPAP usage was higher in Group B. In contrast, congenital pneumonia was higher in Group A, representing improvements in obstetrics and neonatal care in Poland, such as standard testing for group B streptococcus colonization and administration of intrapartum antibiotics. The increase in asphyxia seen in group B may be explained by the introduction of therapeutic hypothermia to the NICU between the two enrollment periods.

An additional interesting observation of this study is the relationship between treatment with nCPAP and the occurrence of PTX. Our finding of a positive relationship between nCPAP use and PTX is similar to some recently published studies [4, 13], whilst others have found no association [16]. We postulate that the increase in PTX that we observed may be due to decreased surfactant administration. Surfactant use has certainly been associated with reduced rates of pneumothorax in ventilated infants, however its role in nCPAP treatment remains to be confirmed and warrants further investigation. In our study we observed the following dependence: the earlier the appearance of pneumothorax, the earlier the day of diagnosis and, at the same time, the shorter the hospitalization, which also indicates the crucial role of etiology. No inflammatory background could be associated with shorter treatment.

In Group B, when LUS was used, the total number of imaging procedures increased, however, since the majority of these were ultrasound investigations, the number of chest X-rays was significantly reduced. Although lung ultrasound examinations are more time-consuming for the clinicians who perform them, they might be considered the stethoscope of the future and have the benefits of reducing radiation and providing the opportunity for real-time imaging. We should note, however, that both pneumopericardium and pneumomediastinum were more often diagnosed in group A, suggesting that LUS may not be sufficient to make these diagnoses [6].

The study confirmed the role of LUS in the diagnosis and monitoring of neonatal PTX. In our unit, the use of this technique enhanced medical care and decreased the number of chest X-rays in newborns with symptomatic pneumothorax. It seems necessary that all neonatologists should be trained in lung ultrasound and LUS should be a self-standing diagnostic method supported by CXR only where the diagnosis is unclear.

### Study limitations

One of the limitations of this study is that it is a retrospective analysis and as a result the risk factors differ between the groups. We believe this did not affect the aims of the study.

## Conclusion

This study demonstrated that implementing LUS as routine care for neonates with PTX reduced the time of diagnosis and decreased the number of the chest X-rays that were used. We strongly advocate lung ultrasound education among NICU personnel as a care necessity in these vulnerable patients.
